# Immunogenicity and Safety of a Novel 13-Valent Pneumococcal Vaccine in Healthy Chinese Infants and Toddlers

**DOI:** 10.3389/fmicb.2022.870973

**Published:** 2022-05-09

**Authors:** Yuliang Zhao, Guohua Li, Shengli Xia, Qiang Ye, Lin Yuan, Hong Li, Jiangjiao Li, Jingjing Chen, Shuyuan Yang, Zhiwei Jiang, Guoqing Zhao, Rongcheng Li, Yanping Li, Jielai Xia, Zhen Huang

**Affiliations:** ^1^Hebei Province Centre for Disease Control and Prevention (Hebei CDC), Shijiazhuang, China; ^2^Shanxi Province Centre for Disease Control and Prevention (Shanxi CDC), Taiyuan, China; ^3^Henan Province Centre for Disease Control and Prevention (Henan CDC), Zhengzhou, China; ^4^National Institutes for Food and Drug Control, Beijing, China; ^5^Walvax Biotechnology Co., Ltd., Kunming, China; ^6^Department of Health Statistics of Air Force Military Medical University (DHSAFMU), Xi’an, China; ^7^Beijing Key Tech Statistical Consulting Co., Ltd., Beijing, China; ^8^Guangxi Zhuang Autonomous Region Center for Disease Control and Prevention (Guangxi CDC), Nanning, China

**Keywords:** pneumococcal conjugate vaccine, sequential test, non-inferiority, superiority, China

## Abstract

**Background:**

To determine the non-inferiority of the seven common serotypes (4, 6B, 9V, 14, 18C, 19F, and 23F) in the 13-valent pneumococcal conjugate vaccine (PCV13) with each serotype conjugated to a tetanus toxoid carrier protein and adsorbed on aluminum phosphate and the superiority of its six additional serotypes (1, 3, 5, 6A, 7F, and 19A) to the serotypes in the PCV7.

**Methods:**

Participants were evenly randomized in a 1:1 ratio into either the PCV13 or PCV7 groups, to receive three doses of the vaccine at the age of 3, 4, and 5 months, respectively, and a booster dose between 12 and 15 months of age. Serotype-specific antibodies were measured using a standardized enzyme-linked immunosorbent assay (ELISA) and opsonophagocytic activity (OPA) microcolony assay method.

**Results:**

A total of 1,040 healthy infants were enrolled. All the seven common serotypes in the PCV13 were non-inferior to those in the PCV7 in terms of the serotype-specific IgG production induced; however, non-inferiority was not shown for serotype 6B after the infant series. The proportion of subjects who reached OPA antibody titers ≥ 1:8 in the PCV13 group was 89.25% or higher. Local reactions and systemic events were mild or moderate in severity and similar between the two groups. No new safety signals were observed.

**Conclusion:**

The newly developed PCV13 was immunogenic for all serotypes and had a comparable safety profile to the marketed PCV7.

## Introduction

The burden of pneumonia remains high worldwide, particularly in developing countries, which is also one of the five main causes of under-five mortality in China, accounting for 60.7% of death (Ministry of Health, People’s Republic of China, 2011). A literature review of published data from 1980 to 2008 showed that pneumococcal disease was a serious health problem in children aged 1–59 months, with serotypes 19F, 19A, and 14 being the most commonly isolated in pneumonia/meningitis patients (Chen et al., 2011). In China, serotypes of 19F, 19A, 23F, 14, and 6B, which cause pneumonia, were the most widely distributed and isolated from children under 14 years (Lyu et al., 2017).

The seven-valent pneumococcal conjugate vaccine (PCV7) (serotypes 4, 6B, 9V, 14, 18C, 19F, and 23F) has been licensed in mainland China since 2008 and covers 79.5 to 88.4% of the prevalent serotypes (Chen et al., 2011). Due to the extensive use of PCV7, the incidence of invasive pneumococcal disease (IPD) has dramatically reduced in children under 5 years (American Academy of Pediatrics Committee on Infectious Diseases, 2010). However, the absolute number of cases of IPD caused by non-PCV7 serotypes, including the 6A, 7F, and 19A serotypes, has increased over time (Conklin et al., 2014). Currently, only one 13-valent pneumococcal conjugate vaccine (PCV13), manufactured by Pfizer, is marketed in China. To address this issue, improve the accessibility of the 13-valent vaccine in infants, and reduce the incidence of disease caused by the common serotypes, a novel vaccine with the same broad coverage as the existing PCV13 is needed. Compared to the PCV7, the novel 13-valent pneumococcal conjugate vaccine (PVC13), thanks to the six additional pneumococcal serotypes (1, 3, 5, 6A, 7F, and 19A) it contains, has the potential to address the major medical and public health issues currently related to pneumococcal disease, by providing broader coverage.

The novel PCV13 was manufactured by Yuxi Walvax Biotechnology Co. Ltd. (Kunming, China), but whether it can provide the same protection and beneficial effects as the PCV7 is still unknown. Therefore, we conducted a randomized, controlled, phase III clinical trial study to determine the immunogenicity, non-inferiority, tolerability, and safety of the PCV13 with respect to the PCV7, in infants aged 3 months. The primary objective of the study was to determine whether the immune responses elicited by the seven common serotypes (4, 6B, 9V, 14, 18C, 19F, and 23F) of the PCV13 were non-inferior to those induced by the serotypes in PCV7 and determine whether the six additional serotype-specific (1, 3, 5, 6A, 7F, and 19A) IgG responses elicited by the PCV13 were adequate, 30 days after the primary series. The secondary objective was to determine whether the immune responses and serum OPA were higher and statistically more significant in PCV13 recipients for the common serotypes.

## Materials and Methods

### Design and Subjects

A randomized, multi-center, double-blinded, and positive-controlled phase III clinical trial study was conducted in three provinces (Henan, Hebei, and Shanxi) of northern China from April 2016 to October 2017 was designed. Three-month-old infants were enrolled and randomly assigned in a 1:1 ratio to receive either the PCV13 or PCV7 at the normal doses at 3, 4, and 5 months of age and a PCV13 booster dose between the ages of 12 and 15 months. Blood samples were collected prior to the injection of the first dose, 30 days after the third dose of the series, before the booster dose, and 30 days after the booster dose.

Eligible subjects were healthy 3-month-old (89–119 days) infants, with a birth weight ≥ 2.5 kg, who had not received a pneumococcal vaccine or any other vaccine 7 days prior to enrollment. The exclusion criteria were as follows: history of invasive disease caused by *Streptococcus pneumoniae*, history of a severe allergic reaction triggered by any vaccines or drugs, history or family history of convulsions, seizures, encephalopathy, or neurological disorders, subjects who had received any blood products (except hepatitis B hyper-immune globulin), and those with any confirmed or suspected diseases, congenital defects, or serious chronic illnesses.

Written informed consent was obtained from each participant’s parent(s) or legal guardian(s). The study was conducted in accordance with the Declaration of Helsinki and Good Clinical Practices (Guidelines, 1996). The study protocol and all relevant study documents and materials were approved by the independent Ethics Committees of the Henan, Hebei, and Shanxi Provincial Centers for Disease Control and Prevention (CDC). This study was registered under the registration number NCT02736240 at Clinicaltrials.gov.

### Vaccines and Administration

The novel PCV13 (batch no. 201511003) was manufactured by Yuxi Walvax Biotechnology Co., Ltd., China. The vaccine was formulated as a full liquid suspension. Vaccines were administered by intramuscular injection in the deltoid muscle or thigh.

The PCV7 was the only pneumococcal conjugate vaccine licensed in mainland China until 2 November 2016 and the study was launched on 2 April 2016 so we took the PCV7 as the control vaccine. The PCV7 (batch no. H29315H13880) was manufactured by Pfizer (NY, United States) and supplied in pre-filled 0.5-ml syringes, with each dose containing 2 μg of pneumococcal polysaccharides of serotypes 4, 9V, 14, 18C, 19F, and 23F and 4 μg of pneumococcal polysaccharide of serotype 6B; each serotype is conjugated to a CRM197 carrier protein and adsorbed on 0.5 mg of aluminum phosphate. As with the PCV7, the novel PCV13 is a single-dose 0.5 ml preparation and contains 2.2 μg of pneumococcal polysaccharides of serotypes 1, 3, 4, 5, 6A, 7F, 9V, 14, 18C, 19A, 19F, and 23F and 4.4 μg of pneumococcal polysaccharide of serotype 6B, with each serotype conjugated to a tetanus toxoid carrier protein and adsorbed on aluminum phosphate.

The PCV7 and PCV13 are not permitted to be concomitantly administered with any other vaccines in China, but other vaccines could be administered after 10 (non-live vaccines) or 14 days (live vaccines) of PCV7 or PCV13 administration. Staff who were responsible for the vaccine storage and administration were not involved in other study processes. The main reason was to reduce the selection bias as the appearance of the two vaccines was similar even by using the blind method.

### Sample Size

The sample size calculation was done by PASS 11.0. For each of the seven common serotypes in both PCV13 and PCV7, based on data from prior studies, we assumed that the percentage of vaccine recipients reaching the serotype-specific IgG concentration threshold of 0.35 μg/ml would be 85%. The total sample size required was 1,040 infants (520 per group) (Supplementary Appendix 1).

### Immunogenicity Assessment

Serotype-specific IgG levels were measured using the standardized enzyme-linked immunosorbent assay (ELISA) method (Wernette et al., 2003). Levels of functional antibodies against each of the 13 serotypes were measured by the serum OPA microcolony assay method (Pohn et al., 2007). OPA titers in the blood samples of 200 subjects, collected from the top 32–36 subjects from each clinical site, were measured, prior to vaccination, 1 month after the third dose, before the booster dose, and 1 month after the booster dose. The serological assays were performed by the National Institutes for Food and Drug Control, which is a member of the WHO Collaborating Center for Standardization and Evaluation of Biologicals, and the methodology is the same as described previously (World Health Organization, 2013).

For each common serotype, the non-inferiority of PCV13 was declared if the lower limit of the two-sided was 97.5% CI for the difference in proportions between two groups (PCV13 vs. PCV7) was ≥ 10%. Alternatively, the non-inferiority criterion was met if the lower bound of the two-sided 97.5% CI on GMC ratio (PCV13/PCV7) was > 0.5. If either of the criteria was met, the non-inferiority would be declared. The proportion of OPA responders and OPA GMT to each serotype was also compared between groups with the method specified for IgG, but the non-inferiority was not necessarily to be tested, for reference purposes only.

For each additional serotype, superiority was demonstrated for the first step if the lower limit of the two-sided 95% CI for the proportion of IgG responders was ≥ 70% and the lower bound of the two-sided 95% CI on the difference in proportion directly compared between groups (PCV13 vs. PCV7) was > 0. The proportion of OPA responders to each serotype was also compared between groups with the method specified for IgG, but the non-inferiority was not necessarily to be tested. The superiority criterion was reached for the second step if the lower bound of the two-sided 95% CI on GMC ratio (PCV13/PCV7 directly) was > 1. The OPA GMTs between groups were compared for reference purposes only, and no hypothesis had been set before. Meanwhile, a *post hoc* analysis was performed to indirectly test the non-inferiority of each of the six additional serotypes in PCV13 with the PCV7 reference serotypes to assess the potential efficacy against the invasive disease of additional serotypes. The reference serotypes were the serotypes among PCV7 with the lowest proportion and the lowest GMC, which were employed to compare the proportion differences and GMC ratios, respectively, using the methods and criteria described above for the common serotypes. As the WHO TRS 977 stated (World Health Organization, 2013), based on the serotype-specific demonstration of efficacy and effectiveness of the 7vPnC vaccine, there is a reasonable rationale for comparing proportions that achieve ≥ 0.35 μg/ml against each serotype that is contained only in the novel vaccine with any serotype in the licensed comparator that achieves the lowest percentage ≥ 0.35 μg/ml.

### Safety Assessments

Subjects were placed under observation for 30 min after vaccination to detect the occurrence of any immediate reactions. Then, solicited adverse reactions occurring after injection were recorded by the parents or legal guardians by filling out a 0–7-day diary card. Following this, parents or guardians filled in contact cards to report any adverse events occurring within 8–30 days of vaccine administration. Finally, serious adverse events (SAEs) occurring within 6 months of the series or booster dose administration were collected through active reporting by guardians and regular follow-up by safety assessors.

Local adverse reactions included pain, nodules, redness, swelling, rash at the inoculation site, and body movement disorders. Solicited systemic adverse events included fever, allergy, headache, fatigue, nausea/vomiting, diarrhea, muscle pain, cough, other discomforts, and other adverse reactions. Safety data, including all expected or unexpected medical events related to vaccination or not, were collected throughout the trial.

### Statistical Analysis

The safety population included all subjects who had received at least one vaccination dose. Comparisons of percentages between the PCV13 and PCV7 groups were made using the Chi-square or Fisher’s exact test.

The primary population for immunogenicity analysis was subjects who had received at least three vaccination doses of the primary series (3, 4, and 5 months) and had valid serum assay results. The secondary population for immunogenicity analysis consisted of children who had completed the primary series, received a booster dose on schedule, and had valid post-booster serum assay results.

As recommended by the WHO (Feavers et al., 2009), the primary immunogenicity endpoint for each pneumococcal serotype was the proportion of subjects with serotype-specific IgG concentrations ≥ 0.35 μg/ml, 1 month after the primary series and the booster dose. The secondary immunogenicity endpoint for each pneumococcal serotype was the proportion of subjects with an OPA titer ≥ 1:8, 1 month after the primary series and the booster dose. To control the family-wise type I error, Bonferroni’s correction was used (Amini and Hochberg, 1995; Watanabe and Takahashi, 2006; Konietschke, 2012; Phillips et al., 2013); thus, a two-sided significance level of 0.025 was adopted for the common serotypes.

To assess the differences in proportions, the two-sided 97.5% (common serotypes) or 95% (additional serotypes) CIs of differences were calculated using the Clopper–Pearson method (Clopper and Pearson, 1934; Newcombe, 1998a,b). For each common serotype, the non-inferiority of PCV13 was demonstrated if the lower limit of the two-sided 97.5% CI for the difference in proportions between the two groups (PCV13 and PCV7) was ≥ –10%. For each additional serotype, superiority was demonstrated, if the lower limit of the two-sided 95% CI for the proportion of IgG responders was ≥ 70% and the lower limit of the two-sided 95% CI of the difference in proportion directly compared between groups (PCV13 and PCV7) was > 0. The proportion of OPA responders for each serotype was also compared between groups by the method specified for IgG, but non-inferiority was not necessarily tested.

For each serotype, IgG antibody concentrations or OPA titers were logarithmically transformed to base 10, and the geometric mean associated with its 95% CI was calculated. To compare GMCs/GMTs between groups, the two-sided 97.5%/95% CIs for GMC/GMT ratios were structured by back-transforming the CIs for means and computed using the t distribution. To calculate GMC, values below the limit of quantitation (BLQ) were halved before analysis (Food and Drug Administration, 2007). For each common serotype, the non-inferiority criterion was met if the lower bound of the two-sided 97.5% CI for the GMC ratio (PCV13/PCV7) was ≥ 0.5. For each additional serotype, the superiority criterion was met, if the lower bound of the two-sided 95% CI for the GMC ratio (PCV13/PCV7 directly) was > 1. The OPA GMTs between groups were compared for reference purposes only, as no hypothesis had been put forth beforehand. The OPA percentages and GMTs between groups were compared for reference purposes only and no hypothesis had been set before.

Additionally, a *post hoc* analysis using the methods and criteria described above for the common serotypes was performed to test the non-inferiority of each of the six additional serotypes in the PCV13 to the PCV7 serotypes that showed the lowest proportion of responders and GMC/GMT.

## Results

### Baseline Demographics and Immune Responses

A total of 1,040 subjects screened from 1,113 infants were enrolled and evenly randomized to the PCV13 or PCV7 group; 503 (96.73%) and 501 (96.35%) subjects in the PCV13 and PCV7 groups, respectively, received the three primary series vaccinations.

**FIGURE 1:**
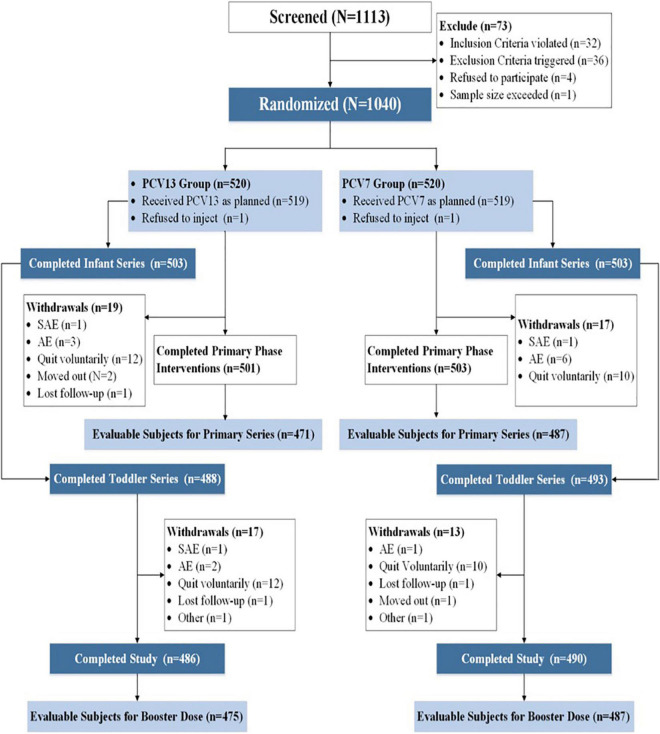
Flowchart for subject enrollment.

A total of 488 (93.85%) and 493 (94.81%) toddlers in the PCV13 and PCV7 groups, respectively, received the booster dose, and 486 (93.46%) and 490 (94.23%) subjects in the PCV13 and PCV7 groups, respectively, completed the study (Figure 1). The main reason for withdrawal was voluntary discontinuation (24 in the PCV13 group and 20 in the PCV7 group). All subjects were Chinese and had similar baseline demographics such as gender, age, height, and weight (Table 1). The proportion of subjects with IgG concentrations ≥ 0.35 μg/ml or IgG GMC for all serotypes at baseline was similar in both the PCV13 and PCV7 groups (*P* > 0.05) (Table 2).

**TABLE 1:** Demographics of subjects.

	PCV13 (*N* = 519)	PCV7 (*N* = 519)	*P*-value
**Age (month)**			
Mean ± SD	3.33 ± 0.30	3.34 ± 0.30	0.4351
**Gender**			
Male, *n* (%)	270 (52.02)	281 (54.14)	0.4939
Female, *n* (%)	249 (47.98)	238 (45.86)	
**Height (cm)**			
Mean ± SD	63.06 ± 2.98	62.82 ± 2.73	0.1680
**Weight (kg)**			
Mean ± SD	7.09 ± 0.90	7.12 ± 0.91	0.6302

**TABLE 2:** Subjects’ antibody-positive rate and geometric mean concentration (GMC) of the pneumococcal capsular IgG levels at baseline.

	Prevaccination					
Serotypes	PCV13	PCV7	*P*-value	PCV13	PCV7	*P*-value
	*N* = 470	*N* = 484		*N* = 470	*N* = 484	
	%	%		GMC	GMC	
**Common**						
4	3.62	2.48	0.3062	0.02	0.02	0.5374
6B	38.51	42.56	0.2026	0.28	0.28	0.9580
9V	17.23	16.32	0.7063	0.08	0.07	0.2285
14	86.17	85.95	0.9219	0.88	0.86	0.7195
18C	16.17	16.12	0.9817	0.09	0.08	0.4948
19F	66.81	68.39	0.6021	0.45	0.48	0.3680
23F	24.47	24.17	0.9156	0.19	0.17	0.4428
**Additional**						
1	10.43	10.33	0.9617	0.11	0.11	0.7657
3	1.70	3.10	0.1596	0.06	0.05	0.1389
5	3.62	4.13	0.6803	0.06	0.06	0.5560
6A	30.64	28.72	0.5164	0.15	0.14	0.4494
7F	17.02	20.04	0.2303	0.12	0.11	0.6474
19A	74.89	76.45	0.5763	0.49	0.51	0.7065

### Immune Response to the Common Serotypes After the Infant Series

In terms of the proportion of responders with anti-pneumococcal polysaccharide antibody levels > 0.35 μg/ml (threshold), non-inferiority was met for all seven serotypes, with the proportion of responders reaching the threshold against the 6B serotype in PCV13 being the lowest (90.45%). The anti-polysaccharide GMC non-inferiority criterion was met for six of the seven serotypes; the lower limit value of the two-sided 97.5% CI for serotype 6B did not meet the non-inferiority criteria (Tables 3, 4).

**TABLE 3:** The proportion of subjects who reached a pneumococcal IgG antibody concentration ≥ 0.35 μg/ml after receiving the infant series, pre-booster, and toddler doses.

Serotypes	After Infant series					Pre-booster		After toddler dose				
	PCV13 *N* = 471	PCV7 *N* = 487	Diff (%) 95%/97.5% CI*	PCV7 Ref.	Diff (%)	PCV13 *N* = 474	PCV7 *N* = 487	PCV13 N = 475	PCV7 N = 487	Diff (%) 95%/97.5% CI*	PCV7 Ref.	Diff (%)
	%	%		%	97.5% CI	%	%		%	%	%	97.5% CI
**Common**												
4	100.0	99.18	0.82 (−0.10,1.74)	NA	NA	74.47	89.73	99.79	99.79	−0.01 (−0.66, 0.65)	NA	NA
6B	90.45	98.97	−8.53 (−11.73, −5.32)	NA	NA	99.58	97.13	100.0	99.79	0.21 (−0.25, 0.67)	NA	NA
9V	99.15	99.79	−0.64 (−1.70, 0.41)	NA	NA	79.11	81.72	100.0	100.0	0.00 (−0.90, 0.92)	NA	NA
14	100.0	99.79	0.21 (−0.25, 0.67)	NA	NA	100.0	100.0	99.79	100.0	−0.21 (−0.68, 0.26)	NA	NA
18C	98.30	97.74	0.56 (−1.45, 2.57)	NA	NA	59.70	76.18	99.79	99.59	0.20 (−0.60, 1.00)	NA	NA
19F	100.0	100.0	0.00 (−0.90, 0.93)	NA	NA	99.16	99.79	100.0	100.0	0.00 (−0.90, 0.92)	NA	NA
23F	97.24	98.56	−1.32 (−3.40, 0.76)	NA	NA	96.41	94.05	100.0	99.79	0.21 (−0.25, 0.67)	NA	NA
**Additional**												
1	99.58	57.49	42.08 (37.65, 46.51)	97.74	1.83 (0.18, 3.49)	87.13	54.41	99.79	91.38	8.41 (5.89, 10.94)	99.59	0.20 (−0.60, 1.00)
3	98.09	45.17	52.91 (48.32, 57.50)	97.74	0.35 (−1.72, 2.42)	55.06	20.12	99.16	92.20	6.96 (4.44, 9.48)	99.59	−0.43 (−1.57, 0.71)
5	98.51	16.63	81.88 (78.40, 85.36)	97.74	0.77 (−1.19, 2.73)	61.18	18.69	97.89	40.04	57.85 (53.31, 62.39)	99.59	−1.69 (−3.31, −0.08)
6A	96.82	78.44	18.38 (14.39, 22.36)	97.74	−0.83 (−3.29, 1.43)	94.73	79.88	99.58	97.74	1.84 (0.40, 3.28)	99.59	−0.01 (−0.94, 0.92)
7F	99.58	24.23	75.35 (71.49, 79.20)	97.74	1.83 (0.18, 3.49)	96.62	46.82	100.0	66.74	33.26 (29.08, 37.45)	99.59	0.41 (−0.24, 1.06)
19A	100.0	97.13	2.87 (1.38, 4.36)	97.74	2.26 (0.75, 3.77)	99.79	96.71	100.0	99.79	0.21 (−0.20, 0.61)	99.59	0.41 (−0.24, 1.06)

**TABLE 4:** Comparison of pneumococcal IgG GMC (μg/ml) after the administration of the infant series, pre-booster, and toddler doses.

Serotypes	After Infant Series					Pre-booster		After toddler dose				
	PCV13 *N* = 471	PCV7 *N* = 487	Ratio 95%/97.5% CI*	PCV7 Ref.	Ratio (%)	PCV13 *N* = 474	PCV7 *N* = 487	PCV13 *N* = 475	PCV7 *N* = 487	Ratio 95%/97.5% CI*	PCV7 Ref.	Ratio (%)
	GMC	GMC		GMC	97.5% CI	GMC	GMC		GMC	GMC	GMC	97.5% CI
**Common**												
4	4.51	7.79	0.58 (0.51, 0.65)	NA	NA	0.57	0.83	3.19	4.27	0.75 (0.66, 0.84)	NA	NA
6B	1.25	3.52	0.36 (0.30, 0.42)	NA	NA	2.08	1.35	10.01	11.70	0.86 (0.75, 0.97)	NA	NA
9V	3.72	4.47	0.83 (0.74, 0.93)	NA	NA	0.59	0.64	4.22	3.87	1.09 (0.97, 1.23)	NA	NA
14	10.38	16.53	0.63 (0.55, 0.72)	NA	NA	3.38	4.83	13.97	17.89	0.78 (0.70, 0.88)	NA	NA
18C	2.91	4.80	0.61 (0.52, 0.70)	NA	NA	0.38	0.51	2.44	3.90	0.63 (0.55, 0.71)	NA	NA
19F	6.95	9.00	0.77 (0.69, 0.86)	NA	NA	2.24	1.78	12.18	7.84	1.56 (1.38, 1.75)	NA	NA
23F	2.93	4.44	0.66 (0.57, 0.76)	NA	NA	1.29	1.11	6.90	10.81	0.64 (0.56, 0.73)	NA	NA
**Additional**												
1	2.15	0.30	7.17 (6.14, 8.37)	3.52	0.61 (0.54, 0.69)	0.71	0.36	2.79	0.67	4.18 (3.76, 4.65)	3.87	0.72 (0.65, 0.80)
3	1.67	0.33	5.02 (4.55, 5.53)	3.52	0.48 (0.42, 0.54)	0.39	0.20	1.89	1.02	1.86 (1.69, 2.04)	3.87	0.49 (0.44, 0.54)
5	1.88	0.05	40.29 (32.02, 50.69)	3.52	0.54 (0.47, 0.61)	0.43	0.17	1.58	0.16	9.63 (8.06, 11.51)	3.87	0.41 (0.37, 0.46)
6A	1.96	0.58	3.38 (2.83, 4.03)	3.52	0.56 (0.48, 0.64)	1.22	0.67	5.82	3.80	1.53 (1.34, 1.75)	3.87	1.50 (1.32, 1.71)
7F	5.47	0.05	105.38 (82.43, 134.72)	3.52	1.56 (1.37, 1.77)	1.29	0.30	4.59	0.31	15.01 (12.58, 17.91)	3.87	1.19 (1.06, 1.32)
19A	3.85	1.06	3.63 (3.25, 4.06)	3.52	1.09 (0.97, 1.23)	1.89	1.15	13.34	3.00	4.45 (4.00, 4.95)	3.87	3.45 (3.07, 3.87)

After the primary series, the proportion of subjects who reached OPA antibody titers ≥ 1:8 against the common serotypes was 89.25% or higher in the PCV13 group and 97.92% or higher in the PCV7 group, which was similar for both groups. Concerning the OPA GMTs, the six common serotypes in the PCV7 elicited higher GMT values than those in the PCV13, except for serotype 19F, which elicited a higher GMT value in the PCV13 group. In addition, GMTs in the PCV13 group were higher than the cutoff value (1:8), and the two groups shared similar trends with respect to the common serotypes (Table 5).

**TABLE 5:** Comparison of pneumococcal OPA antibody levels after the infant series and toddler doses.

Serotypes	Infant series				Pre-booster				Toddler dose			
	PCV13	PCV7	PCV13	PCV7	PCV13	PCV7	PCV13	PCV7	PCV13	PCV7	PCV13	PCV7
	*N* = 93	*N* = 96	*N* = 93	*N* = 96	*N* = 96	*N* = 97	*N* = 96	*N* = 97	*N* = 96	*N* = 97	*N* = 96	*N* = 97
	%	%	GMT	GMT	%	%	GMT	GMT	%	%	GMT	GMT
**Common serotypes**												
4	100.0	100.0	1,227.93	2,291.32	98.96	100.0	439.27	439.39	100.0	100.0	6,182.37	6,208.15
6B	89.25	98.96	528.67	2,928.36	89.58	93.81	220.99	325.63	98.96	97.94	2,661.93	7,564.42
9V	100.0	100.0	1,922.48	1,974.62	97.92	100.0	316.09	305.13	100.0	100.0	6,184.75	5,484.79
14	100.0	100.0	4,511.21	5,594.48	98.96	96.91	1,209.11	1,349.48	100.0	100.0	7,686.96	6,221.93
18C	100.0	98.96	1,356.26	3,129.49	92.71	94.85	68.02	126.85	100.0	98.97	4,173.73	5,507.66
19F	100.0	97.92	652.57	540.56	97.92	91.75	148.23	43.47	100.0	96.91	2,359.26	815.68
23F	95.70	98.96	2,090.78	5,939.64	97.92	97.94	700.00	1,101.39	98.96	98.97	4,943.66	16,131.84
**Additional serotypes**												
1	83.87	0.00	62.13	2.00	68.75	3.09	15.30	2.14	97.92	4.12	321.14	2.28
3	96.77	15.63	511.70	2.80	96.88	44.33	62.20	5.41	97.92	37.11	678.98	5.03
5	98.92	4.17	450.24	2.24	89.58	16.49	56.05	2.96	100.0	5.15	1,375.13	2.61
6A	97.85	80.21	1,079.96	275.54	94.79	76.29	331.48	48.53	95.83	92.78	3,254.75	1,562.59
7F	100.0	37.50	7,741.10	12.76	100.0	36.08	1,024.09	11.86	100.0	52.58	7,244.42	41.47
19A	100.0	44.79	777.70	11.50	88.54	17.53	87.15	3.67	100.0	79.38	5,503.11	72.99

*% Proportion of subjects who reached OPA antibody titers ≥ 1:8.*

### Immune Response to the Additional Serotypes Subsequent to the Infant Series

With regard to the proportion of IgG responders to the six additional serotypes, non-inferiority was shown for all the additional serotypes in the PCV13. In addition, IgG GMCs for the six additional serotypes in the PCV13 group were superior to those in the PCV7 group, with the lower limits of the two-sided 95% CIs being > 1 for all of them (Tables 3, 4).

When compared indirectly with the reference serotypes (18C for proportion and 6B for GMC) in PCV7, the lower limits of two-sided 97.5% CIs for the differences in proportion were all > –10% and lower bounds of two-sided 97.5% CIs for the GMCs ratios were almost > 0.5, with the exception of serotype 6A (0.42) (Tables 3, 4).

With respect to the additional serotypes, PCV13 induced functional antibody responses that were significantly greater than those induced by the PCV7, in terms of both the proportion of responders and OPA GMTs. Except for serotype 1, against which 83.87% of subjects had antibody titers ≥ 1:8 and an OPA GMT of 62.13, the proportion of subjects in the PCV13 group who had OPA antibody titers ≥ was 96.77% or higher, with OPA GMTs of 450.24 or higher.

### Immune Responses to the Common Serotypes After the Booster Dose

For all serotypes common to both vaccines, before the toddler dose, the proportion of subjects who had a pneumococcal IgG concentration ≥ 0.35 μg/ml (threshold) had decreased substantially (Table 3). The toddler dose induced a significant increase in the proportion of IgG responders. The PCV13 and PCV7 both induced higher IgG levels against the seven common serotypes after the toddler dose; GMCs in the PCV13 group were non-inferior to those in the PCV7 group for the respective serotypes (Table 4).

Opsonophagocytic activity antibody responses before the booster dose showed a decreasing trend, in terms of both the proportion of OPA antibody responders and OPA GMTs. For the seven common serotypes, OPA GMTs after the toddler dose showed an upward trend. The difference in GMTs after the toddler dose, and the pre-booster dose or infant series was significant. In addition, OPA GMTs for each of the seven common serotypes in the PCV13 group were similar to those in the PCV7 group (Table 5).

### Immune Responses to the Additional Serotypes After the Booster Dose

The PCV13 was non-inferior to the PCV7 in terms of immunogenicity for all additional serotypes. Moreover, the proportion of subjects who reached antibody threshold concentrations against serotypes 5 and 7F was significantly higher in the PCV13 arm than in the PCV7 arm. All subjects who received the PCV13 booster dose had IgG levels ≥ 0.35 μg/ml against the 7F and 19A serotypes (100%). When compared indirectly with the reference serotype in the PCV7 group (18C with 99.59%), PCV13 was determined as non-inferior (the lower limit of two-sided 97.5% CI > -10%) to PCV7 to all the additional serotypes (Table 3). Moreover, all the OPA responders had OPA antibody titers ≥ 1:8 against the 5, 7F, and 19A serotypes (100%) (Table 5).

After the administration of the infant and toddler series doses, the OPA GMCs against serotype 19A in the PCV13 group were 3.85 and 13.34, respectively. The ratio of the PCV13 7F GMCs to the PCV7 7F GMCs was 15.01, indicating that subjects who received the booster dose may have a higher 7F GMC level (Table 4).

Before the booster dose was given, the OPA GMTs for the additional serotypes were ≤ 100 in all subjects. The OPA GMTs in the PCV13 group were all above 321.14 after administration of the booster dose, which was higher than the levels observed before the administration of the booster dose, with the OPA GMT to the 7F serotype reaching the highest levels. Furthermore, in the subjects who received the booster dose, the magnitude of the increase in OPA GMTs against the 19A serotype after the administration of the booster dose was 60-fold greater than before its administration; this was the highest level reached among all additional serotypes (Table 5).

### Safety and Tolerability

Most local reactions and systemic events were mild or moderate in severity. In most cases, the subjects experienced local reactions related to vaccines within 7 days of the administration of each dose, in both the PCV13 and PCV7 groups. The most commonly reported systemic events were fever and diarrhea. The proportion of subjects who experienced fever with temperatures below 39.0°C was lowest after receiving the booster dose, as compared to the primary series doses. Diarrhea was the most common systemic symptom, with 1.23% of infants manifesting mild and moderate symptoms, respectively. No severe diarrhea cases were observed after the administration of the booster dose. Generally, the most common local reactions were redness and pain, with less than 2.78 and 4.72% of subjects showing minor redness in the PCV13 and PCV7 arms, respectively. In addition, 1.73, 0.79, 0.6, and 0.2% of infants in the PCV13 group experienced only mild pain after the administration of doses 1, 2, 3, and the booster dose, respectively (Table 6).

**TABLE 6:** Percentage of subjects who reported local reactions, systemic events, and other AE after each dose.

Reaction	Dose 1		Dose 2		Dose 3		Booster Dose	
	PCV13 (*N* = 519)	PCV7 (*N* = 519)	PCV13 (*N* = 506)	PCV7 (*N* = 508)	PCV13 (*N* = 503)	PCV7 (*N* = 503)	PCV13 (*N* = 488)	PCV7 (*N* = 493)
	0–7/0–30 days (%)	0–7/0–30 days (%)	0–7/0–30 days (%)	0–7/0–30 days (%)	0–7/0–30 days (%)	0–7/0–30 days (%)	0–7/0–30 days (%)	0–7/0–30 days (%)
**Fever**								
37.1–37.5°C	27.75	32.95	23.12	26.77	22.26	26.04	16.19	18.05
37.6–39.0°C	4.62	8.48	6.52	8.86	9.34	10.54	10.86	9.53
>39.0°C	0.00	0.19	0.20	0.00	0.20	0.20	1.23	0.61
**Diarrhea**								
Mild	5.78	5.01	0.40	1.38	1.19	0.60	1.23	1.01
Moderate	2.70	2.70	2.77	3.15	1.39	1.19	1.23	1.01
Severe	0.39	0.19	0.00	0.00	0.20	0.00	0.00	0.20
**Crying**								
Mild	5.78	4.82	1.78	1.18	0.60	1.59	0.61	0.20
Moderate	2.89	2.50	1.19	1.57	0.60	0.00	0.20	0.20
Severe	0.19	0.00	0.00	0.00	0.00	0.20	0.00	0.20
**Fatigue/sleep increased**								
Mild	4.24	3.66	0.59	0.79	0.60	0.20	0.20	0.20
Moderate	1.35	0.77	0.20	0.00	0.20	0.20	0.00	0.00
Severe	0.00	0.00	0.00	0.00	0.00	0.00	0.00	0.00
**Vomiting**								
Mild	4.24	1.35	0.99	0.79	0.00	0.40	0.82	0.61
Moderate	1.16	1.16	0.00	0.59	0.40	0.00	0.61	0.20
Severe	0.00	0.00	0.00	0.00	0.00	0.00	0.20	0.00
**Cough**								
Mild	3.47	2.50	0.79	1.18	0.80	0.99	0.20	0.61
Moderate	1.54	1.54	0.40	0.79	0.60	0.60	0.61	0.61
Severe	0.00	0.19	0.00	0.00	0.00	0.00	0.00	0.00
**Allergic reaction**								
Mild	0.19	0.19	0.00	0.00	0.00	0.00	0.00	0.00
Moderate	0.19	0.19	0.00	0.20	0.00	0.20	0.00	0.00
Severe	0.00	0.00	0.00	0.00	0.20	0.00	0.00	0.00
**Myalgia**								
Mild	0.19	0.39	0.00	0.00	0.00	0.00	0.00	0.00
Moderate	0.00	0.00	0.00	0.00	0.00	0.00	0.00	0.00
Severe	0.00	0.00	0.00	0.00	0.00	0.00	0.00	0.00
**Redness**								
<15 mm	2.12	4.82	2.37	4.72	2.78	1.99	0.20	0.20
15–30 mm	0.96	1.73	0.40	2.17	1.79	2.39	0.61	0.41
>30 mm	0.00	0.00	0.40	0.20	0.80	0.00	0.20	0.00
**Pain**								
Mild	1.73	1.73	0.79	0.39	0.60	0.20	0.20	0.00
Moderate	0.00	0.00	0.00	0.00	0.00	0.00	0.00	0.00
Severe	0.00	0.00	0.00	0.00	0.00	0.00	0.00	0.00
**Induration**								
<15 mm	0.39	1.73	0.20	0.20	0.00	0.00	0.00	0.00
15–30 mm	0.96	1.16	0.20	0.20	1.19	0.00	0.20	0.00
>30 mm	0.19	0.19	0.40	0.20	0.00	0.00	0.00	0.00
**Swelling**								
<15 mm	0.58	1.54	0.20	1.18	0.20	0.80	0.20	0.20
15–30 mm	0.39	0.77	0.20	0.00	0.40	0.40	0.41	0.20
>30 mm	0.00	0.00	0.00	0.20	0.40	0.00	0.20	0.00
**Pruritus**								
Mild	0.39	0.39	0.00	0.00	0.00	0.00	0.00	0.00
Moderate	0.00	0.00	0.00	0.00	0.00	0.00	0.00	0.00
Severe	0.00	0.00	0.00	0.00	0.00	0.00	0.00	0.00
Other AE*								
Mild	2.31	1.16	1.38	1.97	0.20	0.80	0.20	1.01
Moderate	2.12	2.31	1.58	1.57	1.59	1.59	0.61	0.61
Severe	0.00	0.00	0.00	0.00	0.00	0.00	0.00	0.00

*AE that occurred 0–30 days after vaccination.

Throughout the trial, the incidence of SAEs was similar between the two groups. Fifty-seven SAEs were reported in 41 subjects, with only one SAE in the PCV13 group considered to be related to the study vaccine. Five subjects in the PCV13 group and seven in the PCV7 group withdrew from the study due to AEs (mainly fever and diarrhea). No deaths were recorded during the study.

## Discussion

The widespread use of PCVs in different age groups has significantly reduced the burden of IPD caused by serotypes included in vaccines. With the administration of the PCV7, the incidence of IPD caused by *S. pneumoniae* has significantly decreased (Centers for Disease Control and Prevention [CDC], 2005). With the development of the novel PCV13 vaccine, an assessment of its protection effectiveness in the Chinese infant population has immediately drawn interest. We conducted the non-inferiority study to compare the efficacy of the newly developed PCV13 to that of the PCV7. To some extent, this study provides baseline information on the anti-pneumococcal response to PCV13 serotypes for the first time in Chinese infants, which could further strengthen the epidemiological evidence for its immunogenicity and safety.

This study was conducted to determine whether the novel PCV13 formulation could elicit similar quantitative anti-pneumococcal responses with the PCV7. The most prevalent *S. pneumoniae* strains isolated from 240 Chinese IPDs patients were serotypes 19A (22.1%), 19F (21.7%), and 14 (7.5%) (Zhao et al., 2013). Before vaccination, the anti-pneumococcal responses to these serotypes were significantly high in both groups, which indicated a spontaneous infection in the study population. Aside from serotype 6B, IgG concentrations (≥0.35 μg/ml) and GMCs for all the other common serotypes in the PCV13 were non-inferior to those of the PCV7 serotypes after the infant series. Serotype 6B did not meet the non-inferiority criterion for the lower limit of the two-sided 97.5% CIs, with a proportion below the –10% margin (–11.73%) and GMC less than 0.5 (0.30). One meta-analysis had reported the difference between Pfizer PCV13 and Pfizer PCV7 in 2013. The response rates after a four-dose series for common serotype 6B and additional serotype 3 were lower than for other serotypes, which was consistent with our findings (Ruiz-Aragón et al., 2013). It was also reported that in the immunogenicity of newly developed PCV20, the GMCs of 6B and 3 were lower than those in PCV13 (Senders et al., 2021). However, we cannot completely understand the lower immunogenicity of serotypes 6B and 3. Moreover, the lower limit of the GMC ratio (PCV13/PCV7) for serotype 19F was greater than 1, which indicated the superiority of this serotype in the PCV13, as reported in previous studies (Yeh et al., 2010; Amdekar et al., 2013; Zhu et al., 2016). Concerning the functional OPA responses, with the exception of serotype 19F of the PCV13, which elicited a significantly higher response, PCV13 serotypes elicited similar responses to those of the PCV7 for each common serotype. Overall, these data add to evidence that the novel PCV13 is immunogenic for the common serotypes.

With respect to the six additional serotypes, the PCV13 induced higher serotype-specific IgG and functional OPA antibody levels compared with the PCV7. We found that IgG concentrations (≥ 0.35 μg/ml) 30 days after the primary series in the PCV13 group were higher than those in the PCV7 group. Concerning IgG GMCs, the responses in the PCV13 group were superior to those in the PCV7 group; the GMCs for serotypes 3, 5, and 6A in the PCV13 group were 1.67, 1.88, and 1.96 μg/ml, respectively, and were about fivefold greater than the cutoff value (0.35 μg/ml). In addition, OPA GMTs in the PCV13 group were higher than those in the PCV7 group for all the six additional serotypes, both after the infant series and toddler dose. These responses elicited by the novel PCV13 are similar to those reported in studies on the first-ever PCV13 developed worldwide (Esposito et al., 2010; Togashi et al., 2015). Immune responses to 11 serotypes in the PCV13 increased after the toddler dose compared to those after the infant series, which indicated a good immunological memory.

With regard to the safety profile, PCV13 was well tolerated, and no novel safety signals were observed in this study. Of note, the incidence of fever after each dose was lower in the PCV13 group than in the PCV7 group. Generally, in the PCV13 group, all AEs related to vaccination, regardless of whether they were local or systemic reactions, or other adverse events, were mild or moderate and similar to those in the PCV7 group.

### Strengths and Limitations

This study is the first to provide a baseline for pneumococcal antibodies against serotypes in the PCV13 in infants and not only revealed the prevalence of vaccine-related pneumococcal carriage but also helped assess the clinical efficacy of the novel PCV13. In addition, this study was conducted in three provinces (Henan, Hebei, and Shanxi) of China and involved subjects from diverse ethnicities.

Our study had several limitations. First, it focused on the evaluation of the immunogenicity and safety of a newly developed vaccine in infants. The effects of this novel PCV13 when concomitantly administered with other vaccines specified in pediatric vaccination schedules, such as diphtheria, tetanus, and acellular pertussis (DTaP) vaccine, have not been assessed. Several studies have demonstrated that co-administration of tetanus toxoid-conjugate-containing vaccines, such as MenACYW-TT and Hib-MenCY-TT, with other vaccines, have similar effects on the immunogenicity and safety of any of the vaccines (Esposito et al., 2010; Dhingra et al., 2021). The feasibility of administering the PCV7 concomitantly with the DTaP has been accessed (Li et al., 2008). The immunogenicity of PCV13, co-administered with routine pediatric vaccines, has been widely demonstrated (Amdekar et al., 2013; Diez-Domingo et al., 2013; Togashi et al., 2015). However, the co-administration of PCV and other vaccines was not allowed (Supplementary Appendix 2). We intend to carry out another study to evaluate the feasibility of concomitant administration of the novel PCV13 and other EPI vaccines in order to provide more reliable data and evidence to facilitate clinical use. Second, we did not conduct a parallel comparison between the newly developed PCV13 and the marketed comparator due to study design limitations. We have planned a head-to-head non-inferiority against Pfizer PCV13 in the nearest future, and we will report the results later.

## Conclusion

In summary, the immune responses to the seven common serotypes of the PCV13 were non-inferior to those of the PCV7 serotypes and statistically higher for serotype 19F after the toddler dose. Similar conclusions were drawn after the infant series, with the exception of serotype 6B. With regard to the six additional serotypes in the PCV13, significantly higher immune responses were observed in the PCV13 group, indicating broader protection against pneumococcal disease compared to the PCV7. Furthermore, the novel PCV13 showed similar safety profiles to those of the PCV7. No new safety signals were observed in this study.

## Data Availability Statement

The original contributions presented in the study are included in the article/Supplementary Material; further inquiries can be directed to the corresponding author.

## Ethics Statement

The trial was approved by the Beijing Chaoyang District Centre for Disease Control and Prevention Ethics Committee (the lead institution, IRB No. cYcDPCIRB-20160321-1), Heibei Province Centre for Disease Control and Prevention Ethics Committee (IRB No. 2016-011), Henan Province Centre for Disease Control and Prevention Ethics Committee (IRB No. 2015-YM-004-02), and Shanxi Province Centre for Disease Control and Prevention Ethics Committee (IRB No. SXCDCIRBPJ201600402). Written informed consent to participate in this study was provided by the participants’ legal guardian/next of kin.

## Author Contributions

ZH conceived and designed the study, with input from all other authors. JX provided statistical expertise. SX, GL, and YZ led the clinical team to conduct the study. QY, HL, and JL carried out the laboratory assays and revised the manuscript. ZJ, GZ, and JC analyzed the data. LY and SY interpreted the data. JC and SY drafted the manuscript. All authors had full access to all of the data (including statistical reports and tables) in the study and take responsibility for the integrity of the data and the accuracy of the data analysis, and revised and approved the final version.

## Conflict of Interest

LY, JC, SY, and ZH were employed by Walvax Biotechnology Co., Ltd. ZJ and GZ were employed by Beijing Key Tech Statistical Consulting Co., Ltd. The remaining authors declare that the research was conducted in the absence of any commercial or financial relationships that could be construed as a potential conflict of interest.

## Publisher’s Note

All claims expressed in this article are solely those of the authors and do not necessarily represent those of their affiliated organizations, or those of the publisher, the editors and the reviewers. Any product that may be evaluated in this article, or claim that may be made by its manufacturer, is not guaranteed or endorsed by the publisher.

## References

[B1] Amdekar Y. K. Lalwani S. K. Bavdekar A. Balasubramanian S. Chhatwal J. Bhat S. R. (2013). Immunogenicity and safety of a 13-valent pneumococcal conjugate vaccine in healthy infants and toddlers given with routine vaccines in India. *Pediatr. Infect. Dis. J.* 32 509–516. 10.1097/INF.0b013e31827b478d 23190777

[B2] American Academy of Pediatrics Committee on Infectious Diseases (2010). Recommendations for the prevention of Streptococcus pneumoniae infections in infants and children: use of 13-valent pneumococcal conjugate vaccine (PCV13) and pneumococcal polysaccharide vaccine (PPSV23). *Pediatrics* 126 186–190. 10.1542/peds.2010-1280 20498180

[B3] Amini Y. B. Hochberg Y. (1995). A practical and powerful approach to multiple testing. *J. R. Stat. Soc.* 57 289–300.

[B4] Centers for Disease Control and Prevention [CDC] (2005). Direct and indirect effects of routine vaccination of children with 7-valent pneumococcal conjugate vaccine on incidence of invasive pneumococcal disease–United States, 1998-2003. *MMWR Morb. Mortal. Wkly. Rep.* 54 893–897. 16163262

[B5] Chen Y. Deng W. Wang S. M. Mo Q. M. Jia H. Wang Q. (2011). Burden of pneumonia and meningitis caused by Streptococcus pneumoniae in China among children under 5 years of age: a systematic literature review. *PLoS One* 6:e27333. 10.1371/journal.pone.0027333 22110628 PMC3217934

[B6] Clopper C. J. Pearson E. S. (1934). The use of confidence or fiducial limits illustrated in the case of the binomial. *Biometrika* 26 404–413.

[B7] Conklin L. Loo J. D. Kirk J. Fleming-Dutra K. E. Deloria Knoll M. Park D. E. (2014). Systematic review of the effect of pneumococcal conjugate vaccine dosing schedules on vaccine-type invasive pneumococcal disease among young children. *Pediatr. Infect. Dis. J.* 33 (Suppl 2) S109–S118. 10.1097/inf.0000000000000078 24336053 PMC3944481

[B8] Dhingra M. S. Namazova-Baranova L. Arredondo-Garcia J. L. Kim K. H. Limkittikul K. Jantarabenjakul W. (2021). Immunogenicity and safety of a quadrivalent meningococcal tetanus toxoid-conjugate vaccine administered concomitantly with other paediatric vaccines in toddlers: a phase III randomised study. *Epidemiol. Infect.* 149:e90. 10.1017/s0950268821000698 33814028 PMC8080229

[B9] Diez-Domingo J. Gurtman A. Bernaola E. Gimenez-Sanchez F. Martinon-Torres F. Pineda-Solas V. (2013). Evaluation of 13-valent pneumococcal conjugate vaccine and concomitant meningococcal group C conjugate vaccine in healthy infants and toddlers in Spain. *Vaccine* 31 5486–5494. 10.1016/j.vaccine.2013.06.049 24004465

[B10] Esposito S. Tansey S. Thompson A. Razmpour A. Liang J. Jones T. R. (2010). Safety and immunogenicity of a 13-valent pneumococcal conjugate vaccine compared to those of a 7-valent pneumococcal conjugate vaccine given as a three-dose series with routine vaccines in healthy infants and toddlers. *Clin. Vaccine Immunol.* 17 1017–1026. 10.1128/cvi.00062-10 20427630 PMC2884425

[B11] Feavers I. Knezevic I. Powell M. Griffiths E. (2009). Challenges in the evaluation and licensing of new pneumococcal vaccines, 7-8 July 2008, Ottawa, Canada. *Vaccine* 27 3681–3688. 10.1016/j.vaccine.2009.03.087 19442421

[B12] Food and Drug Administration (2007). *Statistical Review and Evaluation Clinical Studies.* Silver Spring, MD: FDA.

[B13] Guidelines I. G. (1996). ICH Harmonised tripartite guideline: guideline for good Clinical Practice E6(R1). *J. Postgrad. Med.* 47 45–50. 11832605

[B14] Konietschke F. (2012). Multiple testing problems in pharmaceutical statistics. A. Dmitrienko, A. C. Tamhane, and f. BRETZ (2010). Boca Raton: Chapman & Hall/CRC Biostatistics Series. ISBN: 978-1-584 88984-7. *Biom. J.* 54 579–580.

[B15] Li R. C. Li F. X. Li Y. P. Guo S. Y. Nong Y. Ye Q. (2008). Safety and immunogenicity of a 7-valent pneumococcal conjugate vaccine (Prevenar): primary dosing series in healthy Chinese infants. *Vaccine* 26 2260–2269. 10.1016/j.vaccine.2008.02.029 18375021

[B16] Lyu S. Hu H. L. Yang Y. H. Yao K. H. (2017). A systematic review about Streptococcus Pneumoniae serotype distribution in children in mainland of China before the PCV13 was licensed. *Expert Rev. Vaccines* 16 997–1006. 10.1080/14760584.2017.1360771 28745918

[B17] Ministry of Health, People’s Republic of China (2011). *Report on Women and Children’s Health Development in China.* Beijing: Ministry of Health, People’s Republic of China.

[B18] Newcombe R. G. (1998a). Interval estimation for the difference between independent proportions: comparison of eleven methods. *Stat. Med.* 17 873–890. 9595617 10.1002/(sici)1097-0258(19980430)17:8<873::aid-sim779>3.0.co;2-i

[B19] Newcombe R. G. (1998b). Two-sided confidence intervals for the single proportion: comparison of seven methods. *Stat. Med.* 17 857–872. 9595616 10.1002/(sici)1097-0258(19980430)17:8<857::aid-sim777>3.0.co;2-e

[B20] Phillips A. Fletcher C. Atkinson G. Channon E. Douiri A. Jaki T. (2013). Multiplicity: discussion points from the Statisticians in the Pharmaceutical Industry multiplicity expert group. *Pharm. Stat.* 12 255–259. 10.1002/pst.1584 23893876

[B21] Pohn B. Gerlach J. Scheideler M. Katz H. Uray M. Bischof H. (2007). Micro-colony array based high throughput platform for enzyme library screening. *J. Biotechnol.* 129 162–170. 10.1016/j.jbiotec.2006.11.002 17174002

[B22] Ruiz-Aragón J. Márquez Peláez S. Molina-Linde J. M. Grande-Tejada A. M. (2013). Safety and immunogenicity of 13-valent pneumococcal conjugate vaccine in infants: a meta-analysis. *Vaccine* 31 5349–5358. 10.1016/j.vaccine.2013.09.008 24055349

[B23] Senders S. Klein N. P. Lamberth E. Thompson A. Drozd J. Trammel J. (2021). Safety and Immunogenicity of a 20-valent Pneumococcal Conjugate Vaccine in Healthy Infants in the United States. *Pediatr. Infect. Dis. J.* 40 944–951. 10.1097/inf.0000000000003277 34525007 PMC8443440

[B24] Togashi T. Okada K. Yamaji M. Thompson A. Gurtman A. Cutler M. (2015). Immunogenicity and Safety of a 13-Valent Pneumococcal Conjugate Vaccine Given With DTaP Vaccine in Healthy Infants in Japan. *Pediatr Infect Dis J* 34 1096–1104. 10.1097/inf.0000000000000819 26121200

[B25] Watanabe H. Takahashi K. (2006). Points to consider on multiplicity issues in Clinical Trials. *Jpn. J. Biom.* 27 S73–S77. 10.5691/jjb.27.S73

[B26] Wernette C. M. Frasch C. E. Madore D. Carlone G. Goldblatt D. Plikaytis B. (2003). Enzyme-linked immunosorbent assay for quantitation of human antibodies to pneumococcal polysaccharides. *Clin. Diagn. Lab. Immunol.* 10 514–519. 10.1128/cdli.10.4.514-519.2003 12853378 PMC164258

[B27] World Health Organization (2013). *WHO Expert Committee on Biological Standardization:Sixty-Second Report. WHO Expert Committee on Biological Standardization:sixty-Second Report.* Geneva: WHO.

[B28] Yeh S. H. Gurtman A. Hurley D. C. Block S. L. Schwartz R. H. Patterson S. (2010). Immunogenicity and safety of 13-valent pneumococcal conjugate vaccine in infants and toddlers. *Pediatrics* 126 e493–e505. 10.1542/peds.2009-3027 20732948

[B29] Zhao C. Zhang F. Chu Y. Liu Y. Cao B. Chen M. (2013). Phenotypic and genotypic characteristic of invasive pneumococcal isolates from both children and adult patients from a multicenter surveillance in China 2005-2011. *PLoS One* 8:e82361. 10.1371/journal.pone.0082361 24349263 PMC3859574

[B30] Zhu F. Hu Y. Li J. Ye Q. Young M. M. Jr. Zhou X. (2016). Immunogenicity and safety of 13-valent pneumococcal conjugate vaccine compared with 7-valent pneumococcal conjugate vaccine among healthy infants in China. *Pediatr. Infect. Dis. J.* 35 999–1010. 10.1097/inf.0000000000001248 27254028

