# Supplementary material for: Immunogenicity and Safety of a Novel 13-Valent Pneumococcal Vaccine in Healthy Chinese Infants and Toddlers
Source: Front Microbiol. 2022 May 9;13:870973. doi: 10.3389/fmicb.2022.870973 (PMC9125316; doi:10.3389/fmicb.2022.870973)
Supplement: Supplementary file 1 [file Data_Sheet_1.docx]

**eAppendix 1 Sample size calculation.**

The non-inferior margin for the trial would be set at 10%. If the non-inferiority did exist for a proportion of the test group, a sample size of 434 infants per group, 868 total, would provide 80% power to ensure that the lower limit of 97.5% two-sided confidence interval (CI) for difference (test group vs. control group) would be no less than 10%. We also postulated the common variance of serotype-specific IgG GMC, which had been logarithmically transformed by base 10, would not be greater than 1.0. A sample size of 379 infants per group, 758 in total, would have 80% power to detect whether the lower limit of 97.5% two-sided CI for serotype-specific IgG GMC ratio (test group to control group) was above 0.5 or not. To take account of α inflation and β consumption, Bonferroni correction would be employed to α, producing corrected α=0.025/2=0.0125 and corrected power = 1-0.2/7= 0.97. For each of the 6 additional serotypes in PCV13 only: based on the preclinical research, we hypothesized that the proportion of vaccine recipients reaching the serotype-specific IgG concentration threshold of 0.35μg/ml in the test group would be about 80% while that in the control group would not be greater than 50%. Since the percentage of recipients who reaching the threshold of ≥0.35μg/ml to each serotype in PCV13 was approximate 100%, we preferred the GMT value instead of the proportion to evaluate the non-inferiority and superiority of the PCV13 group in toddler dose. Moreover, the percentages experienced a sharp decline before booster dose for almost every serotype, so it was meaningful to care about the rebound post booster. If there was a notable difference between the two groups, a sample size of 274 infants per group, 548 in total, would provide 80% power to demonstrate that the lower limit of 95% two-sided CI for a proportion of the test group would be no less than 70% (the target rate). To control family-wise type II error, power would be adjusted to 0.97 (equals to 1-0.2/6). Theoretically, a total of 868 evaluable participants (434 per group) should enable the trial to achieve the primary outcomes. To take blood draw failures and blood collection refusals into consideration, we would allow for an anticipated 20% dropout rate.

**eAppendix 2 The reasons why the co-administration of PCV and other vaccines was not allowed.**

(i) the package insert of PCV7 clearly forbidden PCV7 be administered concurrently with any other vaccine before 2021, when China FDA changed the package insert. (ii) the systematic reactions following PCV13 or PCV7 would not be clearly considered as the suspected vaccine when they were administered concurrently with other vaccines.
